# Genome sequence of an aflatoxigenic pathogen of Argentinian peanut, *Aspergillus arachidicola*

**DOI:** 10.1186/s12864-018-4576-2

**Published:** 2018-03-09

**Authors:** Geromy G. Moore, Brian M. Mack, Shannon B. Beltz, Olivier Puel

**Affiliations:** 1 Southern Regional Research Center, Agricultural Research Service, United States Department of Agriculture, 1100 Robert E Lee Blvd, New Orleans, Louisiana, 70124 USA; 2 Toxalim (Research Centre in Food Toxicology), Université de Toulouse, INRA, ENVT, INP-Purpan, UPS, Toulouse, France

**Keywords:** *Aspergillus arachidicola*, Genome sequence, Gene ontology, Phylogenomics, Mating-type locus

## Abstract

**Background:**

*Aspergillus arachidicola* is an aflatoxigenic fungal species, first isolated from the leaves of a wild peanut species native to Argentina. It has since been reported in maize, Brazil nut and human sputum samples. This aflatoxigenic species is capable of secreting both B and G aflatoxins, similar to *A. parasiticus* and *A. nomius*. It has other characteristics that may result in its misidentification as one of several other section *Flavi* species. This study offers a preliminary analysis of the *A. arachidicola* genome.

**Results:**

In this study we sequenced the genome of the *A. arachidicola* type strain (CBS 117610) and found its genome size to be 38.9 Mb, and its number of predicted genes to be 12,091, which are values comparable to those in other sequenced Aspergilli. A comparison of 57 known *Aspergillus* secondary metabolite gene clusters, among closely-related aflatoxigenic species, revealed nearly half were predicted to exist in the type strain of *A. arachidicola*. Of its predicted genes, 691 were identified as unique to the species and 60% were assigned Gene Ontology terms using BLAST2GO. Phylogenomic inference shows CBS 117610 sharing a most recent common ancestor with *A. parasiticus*. Finally, BLAST query of *A. flavus* mating-type idiomorph sequences to this strain revealed the presence of a single mating-type (*MAT1–1*) idiomorph.

**Conclusions:**

Based on *A. arachidicola* morphological, genetic and chemotype similarities with *A. flavus* and *A. parasiticus*, sequencing the genome of *A. arachidicola* will contribute to our understanding of the evolutionary relatedness among aflatoxigenic fungi.

**Electronic supplementary material:**

The online version of this article (10.1186/s12864-018-4576-2) contains supplementary material, which is available to authorized users.

## Background

As each new species that is added to *Aspergillus* section *Flavi* is characterized, it seems the numbers of species that are capable of producing B and G aflatoxins are increasing. In the last decade, seven novel B + G producing species have been characterized [1–4]. The potency of B aflatoxins is widely considered to be greater than that of G aflatoxins, but both secondary metabolites, especially aflatoxins B_1_ and G_1_, are regarded as carcinogenic and their purpose or function in nature has yet to be fully understood [5].

*Aspergillus arachidicola* (CBS 117610) was first sampled from an *Arachis glabrata* leaf in the Corrientes province of Argentina, and it was characterized and associated with *Aspergillus* section *Flavi* in 2008 [1]. It has characteristics similar to other section *Flavi* species [1], which may have resulted in repeated misidentifications because it was sampled in the same environment, exhibited similar morphological characteristics, and produced similar mycotoxins to other well-known and characterized species. Since its first association in South America with a single host (peanut plants), *A. arachidicola* has been reported in several hosts/environments such as a Brazil nut shell [6], a clinical sample from a respiratory tract biopsy [7], and a maize kernel [8]. All occurrences of *A. arachidicola* have been reported in similar regions of South America. A North American strain, previously reported as *A. arachidicola* isolated from a diseased alkali bee in 2008 [1], was eventually re-characterized as *A. pseudonomius* by Varga et al. in 2011 [2].

The phylogenetic association and predominating extrolite profile for *A. arachidicola* are similar to *A. parasiticus*, yet it has macro-morphological features (e.g. colony texture and color) that are more similar to *A. flavus* [1]. Interestingly, many of its reported characteristics, such as conidial ornamentation and seriation, were reported to be similar to both *A. flavus* and *A. parasiticus* [1]. Pildain and co-workers’ morphological observations for *A. arachidicola* correlate to a report of hybrid offspring resulting from experimental crosses between *A. flavus* and *A. parasiticus* parent strains, for which many features were considered “*A. flavus*-like, *A. parasiticus*-like or intermediate between the two species” [9]. Other newly characterized species have striking similarities to *A. flavus* and one or more B + G producing species [1–4, 10]. Perhaps *A. arachidicola* is one of several naturally-occurring hybrids to be sampled and characterized as a new species. In this study, we sequenced the genome of this aflatoxigenic fungus, performed genomic comparisons with closely-related aflatoxigenic species, and determined its mating-type to build our understanding of how aflatoxin production has evolved and the evolutionary relationships of aflatoxigenic fungi, especially when reports of phenomena such as inter-specific hybridization and horizontal gene transfer are taken into consideration.

## Results

### Genome information for *A. arachidicola* type strain CBS 117610

Our sequencing coverage for the *A. arachidicola* type strain was ~ 20×. This gave us 4 million reads with a median read length of 352 bp, 674 contigs greater than 1000 bp, and an N50 length of 127,297 bp. Raw sequence reads have been accessioned in the NCBI Sequence Read archive under the number SRR5569329. The *A. arachidicola* genome assembly is 38.9 Mb in size and includes 12,091 protein-encoding genes (Table 1). Additional sequencing quality statistics and predicted genomic information for this type strain are also shown in Table 1. The completeness of the assembly is relatively high as measured by a CEGMA percent completeness score of 95% and a BUSCO percent completeness score of 93% [11].

**Table 1:** Genome characteristics for the *A. arachidicola* Type strain

Genome characteristic	Value
General	
Assembly size (bp)	38,917,187
CEGMA percent completeness	95.56
Percent complete BUSCOs	93.88
G + C (%)	47.9
Protein coding genes	12,091
Protein coding genes > 100 amino acids	11,794
Predicted protein coding sequences > 100 amino acids	
Coding (%)	47.3
Gene density (1 gene every n bp)	3218.6
Median gene length (bp)	1453
Average gene length (bp)	1807
Average number of exons per gene	3.47

### Genomic comparisons with other sequenced aflatoxigenic species

A comparison of genomes was undertaken involving the *A. arachidicola* type strain with those of closely-related aflatoxigenic species. The genome of *A. arachidicola* is most similar to the 39.82 Mb genome of the SU-1 type strain of *A. parasiticus*, which is larger than many other sequenced Aspergilli [12–16]. Since *A. arachidicola* is reported to share similarities with *A. flavus* and *A. parasiticus*, Table 2 includes various morphological, toxigenic and genomic comparisons for these three species, for which there are several observable similarities.

**Table 2:** Morphological, phenotypic and genomic comparison of *A. flavus*, *A. arachidicola* and *A. parasiticus*

Species^a^	Morphology		Phenotype	Genomics			
	Macro^b^	Micro^c^	Toxic SMs^d^	Size (Mb)^e^	Genes^f^	GC (%)^g^	Rep.DNA (%)^h^
*A. flavus* L (NRRL 3357)	55–65 mm; velvety to floccose; olive green	Radiate to columnar; 400–800 μm rf/fr; 20–45 μm gl/el; u/b; 3–6 μm gl/el, sm/fr	B_1_, B_2_, CPA	36.89	13,485	48.22	1.25
	sclerotia (l + v)						
*A. flavus* S (AF70)	55–65 mm; velvety to floccose; olive green	Radiate to columnar; 400–800 μm rf/fr; 20–45 μm gl/el; u/b; 3–6 μm gl/el, sm/fr	B_1_, B_2_, CPA	37.05	13,200	48.30	1.20
	sclerotia (s + n)						
*A. arachidicola* (CBS 117610)	60–65 mm; velvety to floccose; olive to olive brown	Radiate; 250–1000 μm fr; 23–50 μm gl/sg; u/b; 3.5–6.5 μm gl/sg, fr	B_1_, B_2_, G_1_, G_2_	38.92	12,091	47.87	1.65
	sclerotia (a)						
*A. parasiticus* (SU-1)	45–65 mm; velvety to floccose; dark green	Radiate; 250–500 μm fr/rf; 20–35 μm gl/el; u/b; 3.5–6 μm gl, rf	B_1_, B_2_, G_1_, G_2_, OMST	39.82	13,543	47.72	1.43
	sclerotia (o)						

Percentage of repetitive DNA

^a^*A. flavus* and *A. parasiticus* information from Moore et al., 2015; *A. arachidicola* morphology and phenotype information from Pildain et al., 2008

^b^Colony characters on Czpaek’s medium, incubated at 25 °C for 7 days: diameter; texture; color. Sclerotia large and variable in shape (l + v), small and numerous (s + n), elongate (e), occasionally formed (o), or absent/not observed (a)

^c^Conidiophore characters: conidial head; stipe (rough = rf, finely-roughened = fr); vesicle (globose = gl, subglobose = sg, elongate = el); seriation (uniseriate = u, biseriate = b, both/either = u/b); conidia (globose = gl, subglobose = sg, elongate = el, smooth = sm, finely-roughened = fr, rough = rf)

^d^Major toxic secondary metabolites: B and G aflatoxins; cyclopiazonic acid (CPA); O-methylsterigmatocystin (OMST)

^e^Approximate sizes of sequenced genomes

^f^Estimated gene counts based on annotation

^g^GC content for each genome

^h^Percentage of repetitive DNA

### Sclerotium production in *A. arachidicola* type strain CBS 117610

So far, there is no reported evidence that *A. arachidicola* produces sclerotia. Not all strains of *A. flavus* are capable of producing sclerotia [17], while sclerotium production in *A. parasiticus* is said to be an occasional occurrence [18]. Olarte and co-workers [9] reported diminished sclerotium production among their observed *A. flavus* x *A. parasiticus* hybrid offspring. Several genes have been reported to promote development of sclerotia in various fungi, including Aspergilli. For example, *Sclerotinia sclerotiorum ssp1* and *ssp2* orthologs, identified in *A. flavus* and *A. oryzae* as *sspA* and *sspB* [19], were also found in CBS 117610. Another putative sclerotium production ortholog was found that corresponds to the *S. sclerotiorum pac1* gene [20]. Also found in *A. arachidicola* was a putative *sclR* ortholog, originally described in *A. oryzae* [21], as well as *fluP* and *aswA* orthologs, originally described for *A. flavus* [22, 23]. None of these putative orthologs appeared transcriptionally broken in *A. arachidicola*. Even the putative velvet gene (*veA*) homolog in *A. arachidicola*, reported as a regulator of sclerotium development for *A. parasiticus* [24], exhibited 96.5 and 99.5% amino acid sequence identity when compared to *A. flavus* and *A. parasiticus*, respectively.

### Secondary metabolite gene clusters in *A. arachidicola* type strain CBS 117610

Since *A. arachidicola* forms part of a group of species known for aflatoxin production, a primary aim with this genome sequence is to investigate secondary metabolite clusters, specifically those involved in the production of mycotoxigenic compounds. The number of secondary metabolite (SM) clusters within *A. arachidicola* is inferred to be 56 based on analysis using the Secondary Metabolite Unique Regions Finder (SMURF) and 72 based on analysis using the Antibiotics-Secondary Metabolite Analysis Shell (antiSMASH) (Table 3), while closely-related *A. parasiticus* contains 61 and 89 SM clusters according to SMURF and antiSMASH, respectively. The discrepancies observed for SM counts relates to the antiSMASH algorithm, which is designed to predict more than 40 types of gene clusters (e.g., Type 1–3 PKS, NRPS, terpenes, etc.) [25], thus it is considered to provide a more comprehensive list of cluster predictions than SMURF. In contrast to antiSMASH, SMURF conducts cluster predictions for five general SM cluster categories [26].

**Table 3:** Putative secondary metabolite clusters within the *A. arachidicola* and *A. parasiticus* type strains

Backbone type	SMURF*^1^		antiSMASH^2^	
	*A. arachidicola*	*A. parasiticus*	*A. arachidicola*	*A. parasiticus*
NRPS	20	21	25	28
PKS	22	28	21	34
Hybrid PKS/NRPS	3	2	2	2
DMAT	11	10	10	7
Siderophore	N/A	N/A	1	2
Terpene	N/A	N/A	13	16

^1^SMURF *predictions do not include siderophore, terpene or “-like” backbone genes (NRPS-like, PKS-like, NRPS-PKS-like, DMAT-like)

^2^antiSMASH predictions do not include “-like” backbone genes

One very important SM cluster involves the pathway to synthesize the carcinogenic compound known as aflatoxin. The *A. arachidicola* type strain is reported to produce both B and G aflatoxins [1], a genotype confirmed by the absence of the deletion in the *norB/cypA* region. Based on our observations, its aflatoxin gene cluster is similar in size (68 kb) and contains the same 25 genes known to comprise the aflatoxin pathway, in the same orientation, as other B + G producing species, such as *A. bombycis*, *A. nomius* and *A. parasiticus* (Additional file 1: Figure S1) [16]. Another important toxic secondary metabolite is cyclopiazonic acid (CPA), which has been associated with aflatoxin producing species [27], although it was first reported to be produced by *Penicillium cyclopium* [28]. Previous characterization of the CPA biosynthesis cluster for several *A. flavus* strains revealed three genes that are responsible for its production [29]. Although there are no reports of *A. arachidicola* producing CPA, BLAST queries of the nucleotide sequences, and subsequent comparison to the protein sequences, for *A. flavus* (AF36) *maoA*, *dmaT* and *pks-nrps* genes yielded sequence identities (93, 94 and 92%, respectively) within the *A. arachidicola* genome. Its CPA cluster spans 15,918 bp, adjacent to its aflatoxin gene cluster, separated by a genomic distance of 7766 bp. Both the aflatoxin and CPA genomic regions can be found on contig_10 of the *A. arachidicola* genome under GenBank accession number NEXV01000673. The three CPA genes in *A. arachidicola* are also oriented the same as those in the AF36 strain (Additional file 1: Figure S1). A candidate *A. flavus* biocontrol strain known as K49 has a substitution mutation in its *pks-nrps* gene at amino acid 703 that changes a serine (TCA) to a stop codon (TGA) and truncates 3202 amino acids [30]. Closer examination of these genes in *A. arachidicola*, compared to those from the functional CPA cluster in the AF36 biocontrol strain, as well as the non-functional cluster of K49, revealed a deletion mutation within the 1376 bp *dmaT* gene. This single deletion, at nucleotide 474, introduces a frameshift that alters the translation of downstream protein sequence. This shift in the translational reading frame results in generation of a stop codon at nucleotide position 568. Therefore, this shortened *dmaT* protein correlates with loss-of-function (Additional file 1: Figure S1). BLAST query of the CPA nucleotide and amino acid sequences to *A. parasiticus* yielded no evidence of putative homologs, which supports the lack of CPA production in *A. parasiticus*.

No known secondary metabolite gene clusters were predicted that relate to the production of other toxic secondary metabolites reportedly produced by *A. arachidicola* [1], such as aspergillic acid, chrysogine, oryzaechlorin and parasiticolide. First discovered in *Penicillium chrysogenum*, the six genes that are reported to comprise the chrysogine cluster have been very recently accessioned and published for several species, including *A. nomius* [31]. Only five chrysogine genes were reported to exist in Wollenberg’s examined *A. nomius* strain, and these were BLAST queried to the *A. arachidicola* genome. We found all of the putative homologs present on the same contig, and upon assembly they were found to be oriented similarly to those in *A. nomius*. The only exception was the direction of transcription for the *chry6* homolog in *A. arachidicola*, which is the reverse of the *chry6* gene in *A. nomius*. The homolog for the *chry4* gene, reportedly absent in *A. nomius* [31], also could not be located within the *A. arachidicola* type strain genome. The characterization of the putative genes comprising the aspergillic acid cluster of *A. flavus* has yet to be published (Jeff Cary, personal communication), but the putative genes have been deposited in GenBank (accession numbers XM_002373770-XM_002373777). BLAST query of the *A. flavus* gene, and also the respective translated protein, sequences to *A. arachidicola* revealed that all the putative homologs are present on the same contig and are located within a 17,851 bp stretch of nucleotide sequence.

Interestingly, clusters were predicted for several compounds that are most often associated with the genus *Penicillium*, such as citrinin, patulin and penicillin (Table 4), yet these three mycotoxins have also been reported for some Aspergilli [32–34]. A quick BLAST query of patulin cluster genes (based on nucleotide and amino acid sequences from *A. clavatus*) revealed some putative homologs in *A. arachidicola*, but not organized into a cluster. For this reason we cannot support the prediction of the patulin cluster in *A. arachidicola*.

**Table 4:** Known clusters predicted to be shared among closely-related aflatoxigenic *Aspergillus* species

Known cluster	Contig	Location	*A. arachidicola*	*A. parasiticus*	*A. nomius*	*A. flavus* L	*A. flavus* S
4,4′-piperazine-2,5-diyldimethyl-bis-phenol	185	35,897..79024	100	100	100	100	100
Acetylaranotin	21	50,595..95379	9	–	13	13	13
Aflatoxin	10	214,524..301754	44	63	52	56	77
Aflatoxin/Sterigmatocystin	10	214,524..301754	32	59	39	44	81
Aflatrem	91	76,202..99232	62	50	62	75	62
Aflavarin	70	1..57397	100	80	100	100	100
Asperfuranone	15	2472..65958	18	–	18	18	18
Asperipin 2a	38	144,027..173432	75	–	–	100	–
Aspirochlorine	21	50,595..95379	63	–	68	68	59
Azanigerone	49	27,667..120383	8	8	8	8	8
Azaphilone	49	27,667..120383	20	20	20	18	20
Chaetoviridin/Chaetomugilin	15	2472..65958	18	–	18	18	18
Citrinin	197	788..71910	28	28	12	28	28
Cyclopiazonic acid	10	214,524..301754	16	42	–	20	20
Huperzine A	1	642,116..663270	7	–	7	–	–
Mycophenolic acid	373	1..21134	25	–	–	25	–
Notoamide	79	1..57944	11	11	–	11	11
Notoamide/Stephacidin	79	1..57944	11	–	–	–	–
PR toxin	82	54,470..117329	50	–	50	50	50
Patulin	41	75,529..114325	30	13	–	13	13
Paxilline	91	76,202..99232	37	37	37	37	37
Penicillin	193	47,108..71201	12	18	18	18	18
Sirodesmin	21	50,595..95379	9	–	9	9	9
Sterigmatocystin	10	214,524..301754	16	27	21	28	45
Trypacidin	70	1..57397	40	–	40	–	40
Ustiloxin B	54	28,564..88499	78	–	68	–	–
Yanuthone D	15	134,097..256198	20	20	–	–	–

*A. arachidicola* (CBS 117610), *A. parasiticus* (SU-1), *A. nomius* NRRL 13137, *A. flavus* L (NRRL 3357), *A. flavus* S (AF70)

Another predicted cluster in *A. arachidicola* relates to the compound ustiloxin B, which is both a mycotoxin and a phytotoxin that was first discovered in the rice pathogen, *Ustilaginoidea virens* [35]. This particular secondary metabolite has been reportedly produced by *A. flavus* [36], but in our antiSMASH analysis the ustiloxin B cluster was not predicted to exist in *A. flavus* NRRL 3357. However, SMURF has identified the ustiloxin B cluster in NRRL 3357 as cluster #31 (Jeff Cary, personal communication). Whether or not these SM clusters are remnants from a shared ancestor between distant fungi, or if they were inherited through horizontal gene transfer, is unknown but worthy of further studies.

### Secondary metabolite cluster comparisons with other sequenced aflatoxigenic species

There are a number of known secondary metabolite gene clusters that have been reported for aflatoxigenic *Aspergillus* species. “Known” clusters refers to those that antiSMASH outputs as having homology in the GenBank file. A comparison of 57 known clusters, between *A. arachidicola* and its most closely-related species, *A. parasiticus*, revealed 20 of them as predicted to exist in both species (Table 4). Clusters predicted by antiSMASH to exist in only *A. arachidicola* and *A. parasiticus* were those associated with the production of huperzine A and ustiloxin B. Huperzine A is a compound that is being studied for its effectiveness against cognitive decline in elderly patients [37]. There were 10 known clusters predicted to be in *A. parasiticus* that were not predicted for *A. arachidicola*, and seven clusters predicted to exist in *A. arachidicola* were not predicted to exist in *A. parasiticus*. Of those seven predicted not to exist in *A. parasiticus*, five were also found in *A. flavus*. The only other cluster predicted to exist in *A. arachidicola* and one of the other examined species is affiliated with the production of Yanuthone D, a compound with antibiotic and antifungal properties [38], which was also predicted to exist in *A. nomius*. The cluster associated with the production of notoamide and stephacidin compounds was predicted to exist only in *A. arachidicola*. The notoamides and stephacidins are prenylated indole alkaloids that are of interest to biomedical researchers for many potentially beneficial properties that may include anti-tumor, insecticidal and antibacterial properties [39]. One of the predicted clusters with 100% identity to those in the other examined species were those for the production of aflavarin, a metabolite with anti-insectan properties [40] that has recently been associated with sclerotium production in *A. flavus* [23, 41].

### Gene ontology for *A. arachidicola* type strain CBS 117610

For the 691 genes determined to be unique to *A. arachidicola*, among four closely related species (*A. parasiticus*, *A. flavus* L, *A. flavus* S and *A. nomius*), the Fisher’s Exact test showed that 19 of the 32 most significantly-enriched Gene Ontology (GO) terms were associated with molecular function, 12 were associated with biological process, and only one associated with cellular components. However, the 91 sequences associated with the biological process of oxidation reduction were observed having the highest enrichment levels (adjusted *p*-value = 9.19E-4) of all the GO terms/categories (Table 5). The second highest enrichment (second-lowest *p*-value) was shared by sequences related to the molecular functions of oxidoreductase activity (*n* = 23) and heme binding (*n* = 25), both having an adjusted *p*-value of 2.58E-2.

**Table 5:** GO Term enrichment of genes unique to the *A. arachidicola* Type strain

Category	*P*-value^a^	Unique Genes	Total Genes	Term	Ontology^b^
GO:0055114	4.77E-07	91	1218	oxidation-reduction process	BP
GO:0016705	3.30E-05	23	198	oxidoreductase activity, acting on paired donors, with incorporation or reduction of molecular oxygen	MF
GO:0020037	4.01E-05	25	228	heme binding	MF
GO:0009116	0.00010032	10	52	nucleoside metabolic process	BP
GO:0016491	0.00012583	67	945	oxidoreductase activity	MF
GO:0005506	0.00024246	24	240	iron ion binding	MF
GO:0005975	0.00097358	24	264	carbohydrate metabolic process	BP
GO:0003864	0.00607963	2	3	3-methyl-2-oxobutanoate hydroxymethyltransferase activity	MF
GO:0009820	0.01192563	3	11	alkaloid metabolic process	BP
GO:0005247	0.01906088	2	5	voltage-gated chloride channel activity	MF
GO:0006821	0.01906088	2	5	chloride transport	BP
GO:0003824	0.02370096	53	887	catalytic activity	MF
GO:0071949	0.03296192	9	97	FAD binding	MF
GO:0004497	0.03304503	6	53	monooxygenase activity	MF
GO:0005680	0.03766687	2	7	anaphase-promoting complex	CC
GO:0008270	0.03807392	43	717	zinc ion binding	MF
GO:0004553	0.04559701	12	152	hydrolase activity, hydrolyzing O-glycosyl compounds	MF
GO:0003937	0.04578085	1	1	IMP cyclohydrolase activity	MF
GO:0004643	0.04578085	1	1	phosphoribosylaminoimidazolecarboxamide formyltransferase activity	MF
GO:0004731	0.04578085	1	1	purine-nucleoside phosphorylase activity	MF
GO:0006826	0.04578085	1	1	iron ion transport	BP
GO:0006879	0.04578085	1	1	cellular iron ion homeostasis	BP
GO:0008616	0.04578085	1	1	queuosine biosynthetic process	BP
GO:0008887	0.04578085	1	1	glycerate kinase activity	MF
GO:0015099	0.04578085	1	1	nickel cation transmembrane transporter activity	MF
GO:0016428	0.04578085	1	1	tRNA (cytosine-5-)-methyltransferase activity	MF
GO:0018293	0.04578085	1	1	protein-FAD linkage	BP
GO:0031388	0.04578085	1	1	organic acid phosphorylation	BP
GO:0032947	0.04578085	1	1	protein complex scaffold	MF
GO:0033743	0.04578085	1	1	peptide-methionine (R)-S-oxide reductase activity	MF
GO:0035444	0.04578085	1	1	nickel cation transmembrane transport	BP
GO:0051321	0.04578085	1	1	meiotic cell cycle	BP

^a^Over-represented *p*-values

^b^Domains: Biological Processes (BP), Molecular Function (MF), Cellular Component (CC)

### Comparisons of sequenced *Aspergillu*s genomes

**Fig. 1:**
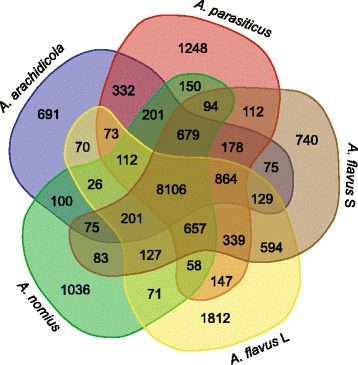
Venn diagram including several species from section *Flavi*. A Venn diagram quantifies unique and orthologous genes for multiple species in section *Flavi*. This diagram of overlapping shapes includes species names, gene counts, and color-shading: CBS 117610 (*A. arachidicola*) is shaded blue, SU-1 (*A. parasiticus*) is shaded red, AF70 (*A. flavus* S) is shaded brown, NRRL 3357 (*A. flavus* L) is shaded yellow, and NRRL 13137 (*A. nomius*) is shaded green

Of its 12,091 predicted genes, orthology analysis (Fig. 1) revealed that *A. arachidicola* shares 8106 genes with four other species examined from section *Flavi*. The lowest number of unique genes shared among more than one species with *A. arachidicola* was 26, and included *A. nomius* and *A. flavus* L. The 691 genes inferred as unique to *A. arachidicola* are the lowest number for this group, since *A. flavus* S (AF70), *A. nomius* (NRRL 13137), *A. parasiticus* (SU-1) and *A. flavus* L (NRRL 3357) harbor more unique genes (790, 1036, 1238 and 1812, respectively). The highest quantity of shared unique genes between *A. arachidicola* and any of the other species examined is with *A. parasiticus* (*n* = 332), which means that both species share the same 332 genes that are not observed in the other examined species. The lowest quantity of shared genes is with *A. flavus* L-type (*n* = 70). Coincidentally, *A. arachidicola* shares 100 genes (the second highest quantity) with the other B + G producing strain examined, *A. nomius*. The number of genes shared among all three B + G species examined, when compared to those shared by all five species, is less than 2.5% (*n* = 201).

**Fig. 2:**
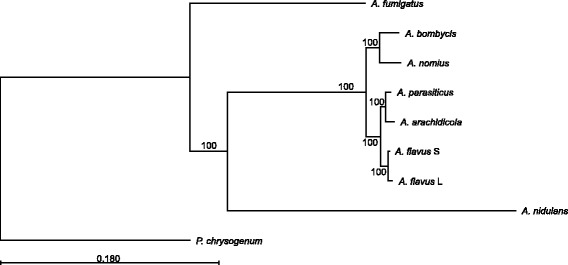
Phylogenomic tree of sequenced *Aspergillus* species. This tree includes inferred patterns of ancestry by phylogenomic comparisons of multiple *Aspergillus* species (*A. fumigatus*, *A. nidulans*, *A. bombycis*, *A. nomius*, *A. arachidicola*, *A. parasiticus*, *A. flavus* L and *A. flavus* S) with *Penicillium chrysogenum* as the outgroup taxa

Phylogenomic comparisons allowed us to infer a species tree for *A. arachidicola* with other *Aspergillus* species, and the outgroup taxa *Penicillium chrysogenum*. Our findings indicate that this species shares a most recent common ancestor with *A. parasiticus* (Fig. 2), which supports the findings of Pildain et al. [1]. This common ancestor of *A. arachidicola* and *A. parasiticus* diverged from the most recent common ancestor of the B-producing morphotypes of *A. flavus*, both of which share a common ancestor. Prior to the speciation event that gives rise to *A. flavus*, *A. parasiticus* and *A. arachidicola* is an event that that split their most recent common ancestor from the predecessor of *A. nomius* and *A. bombycis*.

### *A. arachidicola* CBS 117610 contains a *MAT1–1* idiomorph

**Fig. 3:**
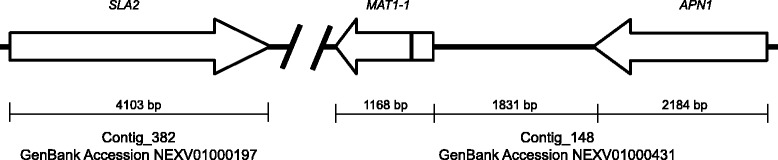
The mating-type locus of *A. arachidicola* CBS 117610. The schematic diagram for its mating-type locus shows the orientation and physical distance shared among the *MAT1–1* gene and the adjacent upstream and downstream genes. The black vertical line in *MAT1–1* represents the approximate location of a 52 bp intron

The *A. arachidicola* type strain contains a single *MAT1–1* gene (Fig. 3), which means this strain is heterothallic (self-infertile). Previous research reported a possible heterothallic existence for most of the species in section *Flavi*, with each species containing a single mating-type idiomorph [42]. The ability of this species to outcross has not yet been reported. Other heterothallic *Aspergillus* species, such as *A. flavus* and *A. parasiticus*, have a mating-type gene flanked by two conserved genes in close proximity: one for DNA lyase (*APN1*) and one for cytoskeleton assembly control (*SLA2*). The flanking of the MAT locus by these genes was recently reported to represent an ancestral configuration in fungi [43]. Although these two genes are consistently found to flank the MAT locus in fungi, the genomic distances separating them may vary [15, 16, 42]. For the *MAT1–1* gene in *A. arachidicola*, the genomic distance to the *APN1* gene was determined to be 1831 bp. Both the *MAT1–1* and *APN1* genes are located on contig_148, which in GenBank has accession number NEXV01000431. The genomic distance separating *SLA2* from the MAT idiomorph could not be determined because the *SLA2* gene was located on a separate contig (contig_382; GenBank accession NEXV01000197) with no overlap. Whether this is because in *A. arachidicola* it is much farther between these genes, or because of a data quality issue, is unknown. The chromosomal location of the mating-type locus in *A. flavus* and *A. parasiticus* is reported to be Chromosome VI [42]. Although it has been reported that the mating-type locus in heterothallic fungi will reside in similar chromosomal locations [44], this has not yet been confirmed for *A. arachidicola*. Comparison of the *A. arachidicola MAT1–1* gene’s amino acid sequence to other *MAT1–1* gene sequences from closely-related species (*A. flavus*, *A. parasiticus*, *A. nomius*, *A. alliaceus* and *A. fumigatus*) revealed 45.5% overall identity, and its identity to both *A. flavus* and *A. parasiticus* was 96% (data not shown). There were only two amino acid substitutions that distinguished *A. arachidicola MAT1–1* sequence from *A. flavus* and *A. parasiticus*, and they were both highly conserved. Of the four substitutions that distinguished *A. arachidicola* and *A. parasiticus* from *A. flavus*, one was highly conserved, one was semi-conserved, and two were non-synonymous. The five substitutions that separated *A. arachidicola* and *A. flavus* from *A. parasiticus* included two highly conserved and three non-synonymous. Across all species examined, 30% of the 371 aligned amino acids showed synonymous substitutions, while 24.5% of amino acid substitutions were non-synonymous.

## Discussion

### Genomic comparisons with other sequenced aflatoxigenic species

Species within section *Flavi* have genomes that are reported to be larger than other aspergilli, which may be the result of gene acquisition [13], or a large percentage of repetitive DNA, as has been suggested for *A. oryzae* [45]. The *A. arachidicola* genome is predicted to contain 1.65% repetitive DNA content (Table 2), which is larger than the other examined species, but does not explain all of the differences in genome sizes. For example, *A. arachidicola* has more repetitive DNA than *A. parasiticus*, but *A. parasiticus* has a 1 Mb larger genome. The reason for *A. parasiticus* and *A. arachidicola* having noticeably larger genomes is unclear, but could relate to either of the aforementioned reasons. Further research is needed to support or refute these possibilities. With regard to the aflatoxin gene cluster, *A. flavus* has a large deletion in the *norB/cypA* region that reportedly prevents synthesis of G aflatoxins [46]. Perhaps the smaller *Aspergillus* genomes are the result of numerous genomic deletions, which do not exist in G-producing species such as *A. parasiticus*. Sequencing the genomes of more B + G aflatoxin producing species may offer more insights into this.

### Sclerotium production in *A. arachidicola* type strain CBS 117610

We examined *A. arachidicola* homologs for several genes that have been reported to regulate sclerotium production in Aspergilli or other fungi, and found that none of them should be non-functional in CBS 117610. Either the true gene associated with sclerotium production has yet to be described, or there are several genes necessary to stimulate sclerotium production in these fungi. Another possibility is that we have yet to determine the environmental conditions necessary to stimulate sclerotium production in this fungus.

### Secondary metabolite gene clusters in *A. arachidicola* type strain CBS 117610

We found an intact AF gene cluster that is oriented, and of similar length/composition, to those of other B + G aflatoxin species. We also found that the clustered genes responsible for the production of CPA are present in this fungus, although a single deletion in one of those genes (*dmaT*) renders the pathway non-functional. If a random recombination event could replace this non-functional *dmaT* gene in CBS 117610 with a functional *dmaT*, then *A. arachidicola* could become an AF and CPA producing species.

Using antiSMASH, we were unable to predict the presence of clustered genes for several mycotoxins reportedly produced by *A. arachidicola*, such as those responsible for the production of chrysogine. Although chrysogine production was reported for some of the sampled *A. arachidicola* strains, Pildain et al. [1] did not specify if CBS 117610 produced this compound. Therefore, any inferences on the genes necessary for chrysogine production in *A. arachidicola* would involve sequencing its relative genomic region in strains or closely-related species that are known to produce it. No complete clusters could be detected via BLAST query and alignment analysis. Since most mycotoxins are the products of gene clusters, it will not be possible to determine the identity of the gene clusters associated with these secondary metabolites until they are experimentally established. Software limitations could also group smaller gene clusters with larger, adjacent clusters. For example, during our antiSMASH analysis the (smaller) CPA cluster was merged with the much larger and adjacent aflatoxin cluster. The antiSMASH output reports the best BLAST hit of any known cluster for each predicted cluster, so the merging of these two clusters initially obscured the presence of the CPA cluster. Other known SM clusters, predicted for other B + G producing species were also predicted to exist in *A. arachidicola*, which were not reported for this species in its first characterization. A complete metabolic profile for *A. arachidicola* will be necessary to determine the presence of any of the compounds reportedly synthesized via these predicted SM clusters.

### Gene ontology for *A. arachidicola* type strain CBS 117610

In our GO analysis, we found the highest levels of enrichment for the biological process of oxidation reduction. Oxidation-reduction reactions (i.e. redox) are reported to correlate with several important facets of fungal biology such as cell differentiation, virulence and growth [47]. Molecular functions for oxidoreductase activity and heme binding were also found to be highly enriched in our GO analysis. Oxidoreductase is an enzyme that is linked with oxidation reduction reactions [48], and heme binding involves fungal acquisition of iron from the host in order to facilitate its survival and growth [49].

### Comparisons of sequenced *Aspergillus* genomes

Our orthology analysis predicted that *A. arachidicola* CBS 117610 contains the least amount of unique genes among the species examined, and that there are a greater number of shared unique genes between *A. arachidicola* and *A. parasiticus*. The more aflatoxigenic species are examined and included in orthology analysis, the likelihood increases of discerning genes that could improve species delimitation via diagnostic PCR. What has yet to be determined is whether or not “unique” genes are wholly unique. For example, if the shuffling of genetic material (i.e. recombination) results in genes that are a composite from two or more different genomes, then orthology analysis might consider them unique when they are merely lacking identity with other examined species. More thorough comparisons of the unique genes in sequenced genomes of closely related species will either support or refute this. Alternatively, these unique genes could relate to certain gene clusters that are found in *A. arachidicola* but not in other closely related species. Our phylogenomic inferences support previous reports that *A. arachidicola* and *A. parasiticus* share a most recent common ancestor [1]. This may correlate with their sharing the greatest number of unique genes. Phylogenomics involving even more aflatoxigenic species will better refine our understanding of the evolution of toxic secondary metabolite clusters, and offer insights regarding the potential impacts of recombination on these clusters within mycotoxigenic *Aspergillus* species.

### *A. arachidicola* CBS 117610 contains a *MAT1–1* idiomorph

We found that this strain has the *MAT1–1* mating-type, which could account for a lack of sclerotium production in the *A. arachidicola* type strain. It remains to be seen whether the other sampled *A. arachidicola* isolates are of the same mating type. According to Horn et al. [50], there may be roles affiliated with each mating type, and that strains capable of producing conidia and sclerotia are hermaphroditic. If conidia provide the donor (i.e. paternal) genetic material, and sclerotia provide the receptor (i.e. maternal) genetic material, then perhaps CBS 117610 is a true “male” strain since it fails to produce sclerotia. The mating types of other sampled *A. arachidicola* isolates, specifically *MAT1–2* strains, would need to be characterized, and additional comparisons made, to support or refute this. Further, if no *A. arachidicola* strains are found that produce sclerotia, perhaps inter-specific mating experiments involving *A. arachidicola* conidia and sclerotia from closely related species (*A. flavus* or *A. parasiticus*) might reveal its reproductive potential. Experimental crosses involving *A. flavus* and *A. parasiticus* proved that inter-specific hybridization is possible [9].

## Conclusions

*Aspergillus arachidicola* is one of several recently-characterized fungi that seems to share morphological, genomic and chemotype similarities to several other historical *Aspergillus* species. It contains several secondary metabolite gene clusters (functional as well as non-functional) that warrant further study. Obviously, there is more research required to support or refute the potential for *A. arachidicola* to be a naturally-occurring hybrid species. For example, there would need to be more *A. arachidicola* isolates examined. Other than the type strain, only six were reported by Pildain et al. [1]. However, there have been few additional reports of *A. arachidicola* isolates sampled [6–8]. Hybrid organisms can suffer fitness disadvantages for adaptability to certain niches, based on inherited traits from either parent [51]. Inter- and intra-specific mating experiments would need to be undertaken to determine the fecundity of *A. arachidicola*, since it has been reported that hybrid offspring often suffer infertility [9, 51]. And thorough comparisons (e.g. SM production) would need to be made between the inter-specific hybrids reported by Olarte et al. [9] and multiple *A. arachidicola* strains. Given the increasing numbers of reports that highlight sexual potential (both intra- and inter-specific) of fungi, as well as evidence of horizontal gene transfer, it is important not to discount the potential impacts these phenomena may have on speciation and the evolutionary relatedness of fungal organisms.

## Methods

### Genome sequencing and annotation of *A. arachidicola* CBS 117610

The CBS 117610 genome was sequenced using a Personal Genome Machine (PGM) from Life Technologies (Grand Island, New York). A loopful of CBS 117610 spores were inoculated in Yeast Extract Sucrose (YES) liquid medium (Sigma-Aldrich, Saint Quentin-Fallavier, France), and kept in agitation in an orbital incubator at 170 rpm, at 27 °C, for five days. DNA extraction was performed by grinding a portion of mycelium in a 5 ml mortar on ice, followed by its addition to 5.5 ml lysis buffer (20 mM Tris-HCl, 250 mM NaCl, 25 mM EDTA 0.5 M pH = 8, 1% SDS). The content was transferred to a 15 ml tube and 12.5 μl of Proteinase K (20 mg/ml) (EMD Millipore, Billerica, MA) were added, then the samples were incubated for 30 min up to 1 h at 37 °C, followed by an additional 10 min at 65 °C. Thereafter, one volume of phenol/chloroform (7:3, v:v) was added, and samples were vigorously shaken and centrifuged at 3080 x g for one hour. Supernatant was recovered into a new tube, and 6 μl RNAse A (100 mg/ml) (Serva electrophoresis GmbH, Heidelberg, Germany) were added prior to incubation for 2–3 h at 37 °C. Next, one volume of chloroform was added and centrifuged at 3080 x g for 10 min. Supernatant was recovered into a new tube, and one volume of isopropanol was added. At this point, samples were softly shaken for 2 h in a horizontal shaker and kept overnight at 4 °C. The next day, samples were centrifuged at 13,000 x g for 30 min. The supernatant was eliminated and the pellet carefully washed with 300 μl of 70% ethanol, then centrifuged at 13,000 x g for 15 min, followed by a gentle aspiration of the supernatant. Finally, the pellet was re-suspended with 30 μl of pure water. DNA samples were quantified using a NanoDrop ND-1000 (NanoDrop Technologies, Wilmington, DE, USA). Library preparation and sequencing were conducted according to previously reported protocols [15, 16]. A total of 4.1 million reads were used for genome assembly with SPAdes (version 3.9.0) [52]. BUSCO (version 1.22) [11] was used to train Augustus with ortholog information from the “fungi” lineage. Annotation software pipelines such as MAKER [53], GeneMark [54], Augustus [55], as well as the Swiss-Prot database for detecting protein homology, were used as previously described [15, 16]. A transcriptome assembly from *A. parasiticus*, made using Trinity, was also used as EST evidence within MAKER. We then used NCBI’s Genome Annotation Generator [https://github.com/genomeannotation/GAG] for annotation format conversion, and deposited the annotation under BioProject project number PRJNA385482.

### Genomic comparisons among *Aspergillus* species

The antiSMASH [27] and SMURF [28] programs were used to predict SM clusters in *A. arachidicola*. Phylogenomic analysis was performed by detecting orthologous proteins within other fungi using Proteinortho (version 5.13) [56], aligning them using MUSCLE (version 3.8.31) [57], and concatenating them into a 2 Mb amino acid alignment using GBLOCKS (version 0.91) [58]. We inferred our phylogenetic tree using RAxML-HPC (version 8.1.17) [59] using data and conditions as previously described [15, 16]. Gene Ontology term enrichment was performed using goseq (version 1.28.0) [60] using the “hypergeometric” option. Repetitive elements were identified using RepeatMasker (version 4.0.7) [61] with the RepBase [62] library and species set to fungus.

The sclerotium-related genes involved BLAST queries of nucleotide and amino acid sequences to those of *A. arachidicola*. All SM gene cluster comparisons involved BLAST queries to the *A. arachidicola* genome, then they were aligned to its contig sequences for distance mapping. Similarly, the mating-type (MAT) locus comparisons were performed by BLAST query of *A. flavus MAT*, *APN* and *SLA* genes to the *A. bombycis* genome. Distance mapping between the examined genes/clusters were performed using Sequencher software (Gene Codes Corporation, Ann Arbor, MI).

## Additional file

Additional file 1: **Figure S1.** Comparison of aflatoxin and CPA gene clusters for *A. arachidicola* and several aflatoxin B + G species’ type strains**.** The schematic diagram (A) shows the orientation and relative sizes (bp) of genes in the aflatoxin gene cluster of *A. arachidicola* (CBS 117610; red), *A. parasiticus* (SU-1; green), *A. nomius* (NRRL 13137; blue) and *A. bombycis* (NRRL 26010; purple). Panel B shows the orientation and relative sizes (bp) of genes in the CPA gene cluster of *A. arachidicola* (CBS 117610; red), *A. nomius* (NRRL 13137; blue) and *A. bombycis* (NRRL 26010; purple). The *A. parasiticus* type strain did not contain a cluster of CPA genes. Panel C shows the orientation and distance (bp) separating the aflatoxin and CPA gene clusters in *A. arachidicola* (CBS 117610; red). The respective gene clusters and their distances, in *A. nomius* and *A. bombycis*, were not found to share the same contig; therefore, they could not be determined. (PDF 587 kb)

## Abbreviations

antiSMASH: Antibiotics & Secondary Metabolite Analysis Shell
APN1: DNA lyase gene
BLAST: Basic local alignment search tool
bp: Base pairs
BUSCO: Benchmarking Universal Single-Copy Orthologs
CBS: Centraalbureau voor Schimmelcultures
CEGMA: Core Eukaryotic Genes Mapping Approach
CPA: Cyclopiazonic acid
GO: Gene Ontology
JCVI: J. Craig Venter Institute
JGI: Joint Genome Institute
MAT: Mating-type
NCBI: National Center for Biotechnology Information
NRPS: Non-ribosomal peptide synthase
NRRL: Northern Regional Research Laboratory
OMST: *O*-methylsterigmatocystin
PCR: Polymerase Chain Reaction
PGM: Personal genome machine
PKS: Polyketide synthase
RAxML: Randomized axelerated maximum likelihood
SLA2: Cytoskeleton assembly control gene
SM: Secondary Metabolite
SMURF: Secondary metabolite unique regions finder
