# Supplementary figures and images for: Genome sequence of an aflatoxigenic pathogen of Argentinian peanut, Aspergillus arachidicola
Source: BMC Genomics. 2018 Mar 9;19:189. doi: 10.1186/s12864-018-4576-2 (PMC5845213; doi:10.1186/s12864-018-4576-2)

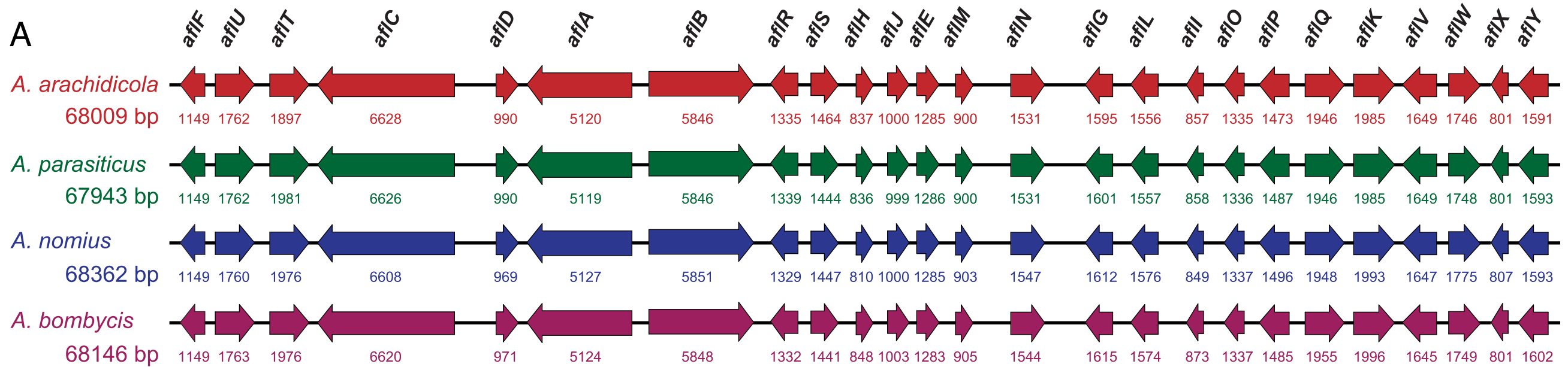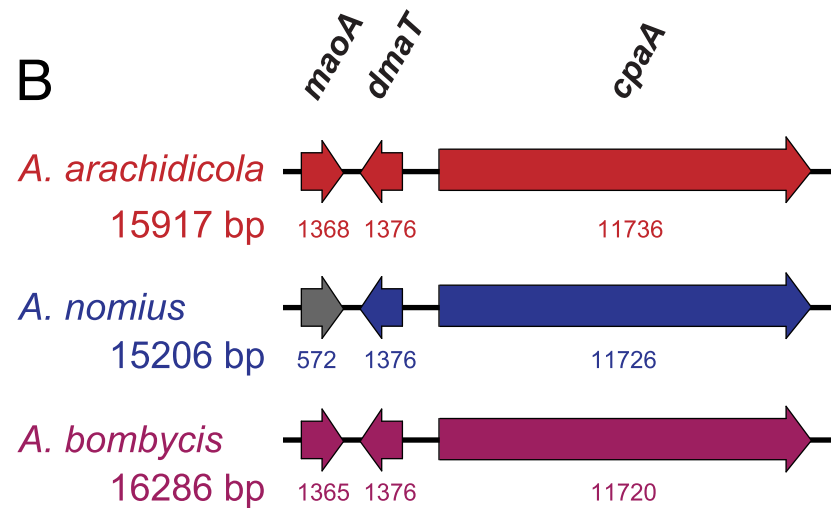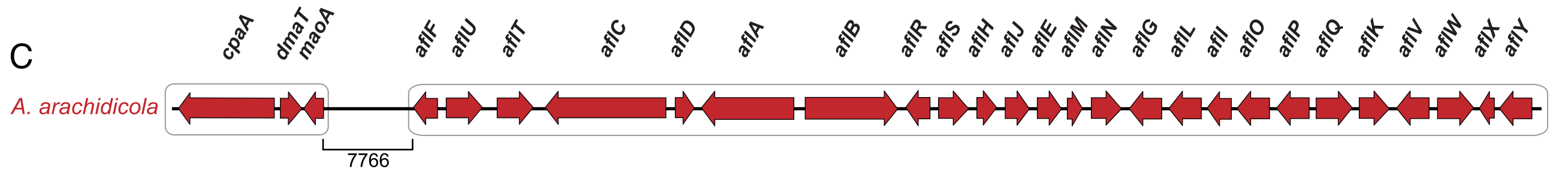

Contig\_10; GenBank accession NEXV01000673

Supplement: Supplementary file 1 — Figure S1. Comparison of aflatoxin and CPA gene clusters for A. arachidicola and several aflatoxin B + G species’ type strains. The schematic diagram (A) shows the orientation and relative sizes (bp) of genes in the aflatoxin gene cluster of A. arachidicola (CBS 117610; red), A. parasiticus (SU-1; green), A. nomius (NRRL 13137; blue) and A. bombycis (NRRL 26010; purple). Panel B shows the orientation and relative sizes (bp) of genes in the CPA gene cluster of A. arachidicola (CBS 117610; red), A. nomius (NRRL 13137; blue) and A. bombycis (NRRL 26010; purple). The A. parasiticus type strain did not contain a cluster of CPA genes. Panel C shows the orientation and distance (bp) separating the aflatoxin and CPA gene clusters in A. arachidicola (CBS 117610; red). The respective gene clusters and their distances, in A. nomius and A. bombycis, were not found to share the same contig; therefore, they could not be determined. (PDF 587 kb) [file 12864_2018_4576_MOESM1_ESM.pdf]
